# The Impact of Acute EBV Infection on Changes in the Serum Proteome in Children—A Pilot Study

**DOI:** 10.3390/pathogens13060471

**Published:** 2024-06-04

**Authors:** Katarzyna Mazur-Melewska, Magdalena Luczak, Joanna Watral, Paweł Małecki, Anna Mania, Magdalena Figlerowicz

**Affiliations:** 1 Department of Infectious Diseases and Child Neurology, Karol Marcinkowski University of Medical Sciences, 60-572 Poznań, Poland; 2 Department of Biomedical Proteomics, Institute of Bioorganic Chemistry, Polish Academy of Sciences, 61-704 Poznań, Poland

**Keywords:** Epstein–Barr virus, proteomics, children

## Abstract

This study investigates the impact of Epstein–Barr virus (EBV) infection on children’s proteomes across different phases of the disease, utilising seventy-nine blood samples categorised into three groups: EBV-naive patients, acute infectious mononucleosis (IM) cases, and convalescents followed up for 12 months post-IM. The aim is to identify proteins influenced by EBV infection, shedding light on the chronic processes triggered by the virus. The results reveal thirty-nine proteins distinguishing between naive patients and those with IM, including actin, lumican, peroxiredoxin-2, fibulin-1, gelsolin, and alpha-2-macroglobulin, which are involved in immune responses, cell adhesion, and inflammation. Elevated oxidative stress markers like peroxiredoxin-2 in IM patients suggest potential links to EBV’s induction of reactive oxygen species. Increased levels of apolipoproteins A-I, A-IV, C-IV, and M during IM imply associations with viral infection, while complement system proteins (C1q, C1r, and C8 gamma chain) are also elevated, reflecting their role in the immune response and viral clearance. This study’s focus on children provides unique insights into EBV’s impact on young populations, emphasising proteomics’ role in uncovering protein associations and understanding the virus’s long-term consequences. However, specific relationships between identified proteins and EBV infection require further investigation.

## 1. Introduction

Epstein–Barr virus (EBV) is a member of the gamma-herpes virus family, causing widespread infections in both children and adults across the globe. Following transmission through saliva, the virus undergoes replication within the epithelial cells and subsequently infects tonsillar B cells, leading to a lifelong infection. This infection progresses from an acute phase to a persistent phase with intermittent exacerbations [1]. EBV lytic reactivation is strategic to the virus life cycle and to most EBV-related diseases [2].

Primary infection with EBV takes the form of infectious mononucleosis (IM), which may be presented with lymphadenopathy and hepatosplenomegaly of varying severity, accompanied by fever. In some patients, the disease is asymptomatic, although there are also cases of neurological periods of acute symptoms [1] and gastrological [2] and cardiological complications [3]. The EBV-induced process is characterised by T-cell hyperproliferation with a strong CD8^+^ response [4]. The severity of which, next to viral load, is a confirmed contribution to the severity of infection symptoms. After the primary infection, EBV can become a causative factor of neoplastic processes. The current EBV is involved in the oncogenesis of over 200,000 cancer cases annually [5]. EBV is the first virus to show an oncogenic effect in humans when isolated from lymphoblasts cultured from Burkitt’s lymphoma. Further observations confirmed the importance of EBV in the development of a number of neoplastic diseases, e.g., Hodgkin’s lymphoma, diffuse large B cell lymphoma, nasopharyngeal carcinoma, gastric carcinoma, and post-transplant lymphoproliferative disease [6]. Most of the distant diseases are associated with the transition of EBV infection from an acute to a latent form, which significantly affects the immunology of the infected person [7]. Immunological and virological causes that lead to oncological complications, among others, remain in the sphere of constant interest. It is particularly important that these complications affect a relatively small group of people [6].

Proteomics can provide insights that genomics or traditional laboratory techniques cannot explain, such as protein–protein interactions and the presence or modulation of post-translational modifications [8]. The application of proteomics in EBV research is of interest to some scientists. Thus far, methods based on RNA sequencing have allowed for the assessment of both viral transcription and the changes occurring in lymphoid cells. However, post-transcriptional modifications, which can significantly alter the host’s proteome, remain poorly understood [9]. Proteomic studies on the EBV virus have primarily focused on the acute phase of infection, with findings serving as comparative material for research on other conditions, primarily hemophagocytic lymphohistiocytosis [10]. Limited knowledge exists regarding the distant post-transcriptional consequences of infection. As demonstrated by Deyfus et al., contracting IM leaves behind a proteomic “molecular fingerprint,” the understanding of which could potentially be beneficial in the early diagnosis and monitoring of EBV-related diseases, mainly of autoimmune origin [11].

The objective of our study was to conduct proteomic analysis on plasma samples from children infected with EBV during the acute, full-blown, and convalescent phases of the disease. This assessment aimed to identify the proteins influenced by EBV infection, serving as evidence for the development of a chronic process. Our particular emphasis on a demographic of children with a notably high prevalence of EBV infection contributes to broadening our understanding of the virus’s effects on adolescents.

## 2. Materials and Methods

### 2.1. Plasma Sample Collection

As part of the study, 79 blood samples were collected from 56 children aged 1 to 18 years (mean age: 8.78 ± 3.18 years; 23 girls and 33 boys) under the care of the Department of Infectious Diseases and Paediatric Neurology and the Hepatology Outpatient Clinic in Karol Jonscher Hospital University of Medical Sciences in Poznań. We collected blood samples from patients at three different disease stages as follows: naive patients; the acute phase of the symptomatic IM, when the patients were hospitalised in the infectious disease ward; and 12 months after discharge, from children, followed up in the outpatient clinic. 

#### 2.1.1. The Tested Material Was Divided into Three Research Groups

EBV-1, (n = 33): the group included material obtained from healthy patients who did not present with symptoms of IM in the physical examination and whose blood was excluded from anti-EBV antibodies: viral capsid antigen class IgM (EBV-VCA IgM)-negative, viral capsid antigen class IgG (EBV-VCA IgG)-negative, Epstein–Barr nuclear antigen (EBNA IgG)-negative. Patients in the group were recruited among children hospitalised for neuroimagination examinations due to chronic headaches. The additional conditions for qualifying for the control group were as follows: The exclusion of developing infection, including clinical observation of the patient supplemented by a daily paediatric examination (conducted during the 3-day stay of the diagnostic patient in the ward) and routinely performed during hospitalisation tests, C-reactive protein, morphology with a smear, erythrocytes, leucocytes, and activity of the liver enzymes (alanine and aspartic aminotransferase and gamma-glutamyl transpeptidase) used in the qualification for diagnostic procedures under general anaesthesia. The exclusion criteria for the EBV-1 group comprised the following: a positive result of a paediatric physical examination indicating infection development in the child, obtained during a three-day observation and/or elevated markers of inflammation and/or increased liver enzyme activity.

EBV-2, (n = 23): Material from patients presenting symptoms of acute IM. EBV infection was diagnosed based on typical clinical symptoms, which were the reason for admission to the hospital, such as high fever, lymphadenopathy, hepatomegaly, and splenomegaly, and the presence of EBV-VCA IgM antibodies in their serum. The EBV IgG and EBNA IgG were negative. For research purposes, samples were collected only from patients who came to the ward without prior antiviral treatment.

EBV-3 (n = 23): After IM, the children from group EBV-2 were observed over the next 12 months. During this observation, the resolution of the clinical symptoms of the acute process was confirmed. Positive EBV IgG antibodies and negative EBV IgM were found in the blood of these patients. The blood samples, qualified as EBV-3, were collected from these children after 12 months from the acute phase (Table 1). 

Patients with congenital immunodeficiencies and patients undergoing immunosuppressive therapies were excluded from the analysis. Also, the patients with a history of *Cytomegalovirus* (CMV), hepatitis B virus (HBV), hepatitis C virus (HCV), human immunodeficiency virus (HIV) and *Toxoplasma gondi* infections were excluded from the study. 

#### 2.1.2. Clinical Evaluation

A specialist in paediatrics and infectious diseases conducted the clinical examination of the patients. The patients were assessed at the time of admission to the infectious diseases ward for the presence of symptoms of mononucleosis syndrome.

### 2.2. Biochemical Research

Serological tests for antibodies specific to EBV antigens were performed in the Central Laboratory of the Karol Jonscher Hospital in Poznań. 

### 2.3. Serological Tests

The antibody assays were based on conventional indirect immunofluorescence, conducted using the manual system in the Central Laboratory of the Karol Jonscher Hospital in Poznań. 

### 2.4. Proteomics

Proteomic studies were conducted at the European Centre for Bioinformatics and Genomics, Institute of Bioorganic Chemistry, Polish Academy of Sciences. The protein concentration was measured using a commercial BCA kit (Pierce). As previously described, ten micrograms of protein were reduced, alkylated, and digested with trypsin [12]. Each sample was prepared for digestion in duplicate. For each run, 1.5 μg of the protein digest was subjected to nano-LC–MS/MS (nano-liquid chromatography–tandem mass spectrometry) analysis using a Dionex UltiMate 3000 RSLC and Q-Exactive Orbitrap mass spectrometer (Thermo Fisher Scientific, Waltham, MA, USA) as previously described [12]. After each LC–MS/MS run, the raw files were analysed by Proteome Discoverer v. 1.4.14 (Thermo Fisher Scientific, Waltham, MA, USA).

### 2.5. Statistics

The statistical assay was performed in STATISTICA (version 13, TIBCO Software Inc., Palo Alto, CA, USA). Data were expressed as the mean ± standard deviation (SD). Student’s *t*-tests were used to compare the continuous variables, where appropriate. Differentially expressed proteins (*p*-value < 0.05, fold change > 1.4) were subjected to the analysis in Perseus 1.4.1.3 software [13,14]. Proteins were annotated according to their gene ontology in the biological process category and then were subjected to enrichment analysis using the right-tailed Fisher’s exact test. Multivariate analyses were conducted by untargeted principal component analysis (PCA) and hierarchical clustering. Correlations between EBV-1 and EBV-2 and EBV-1 and EBV-3 were performed using the Pearson correlation test. A *p*-value of <0.05 was considered statistically significant. 

## 3. Results

A total of 544 proteins have been identified. The reproducibility between the biological and technical replicates was checked using scatter plots and correlation analysis. The Pearson correlation coefficients for technical repeats ranged between 0.88 and 0.94, while for the biological repeats, between 0.82 and 0.92.

**Figure 1:**
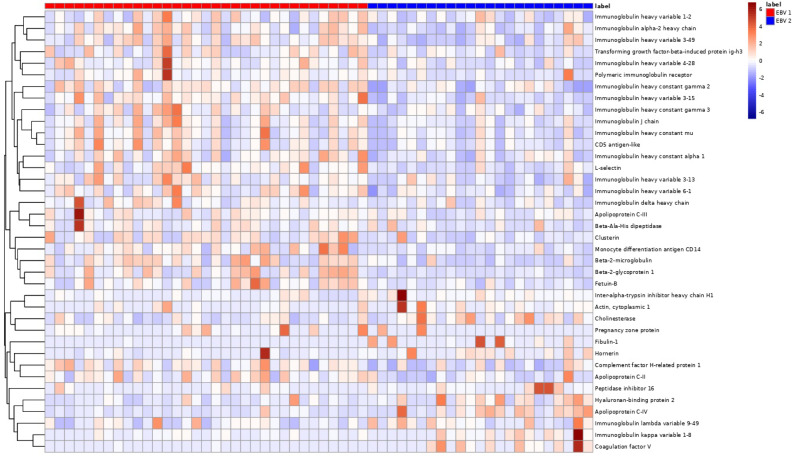
Hierarchical clustering of proteins (N = 56 patients; K = 38 proteins), comparing groups EBV-1 vs. EBV-2.

**Figure 2:**
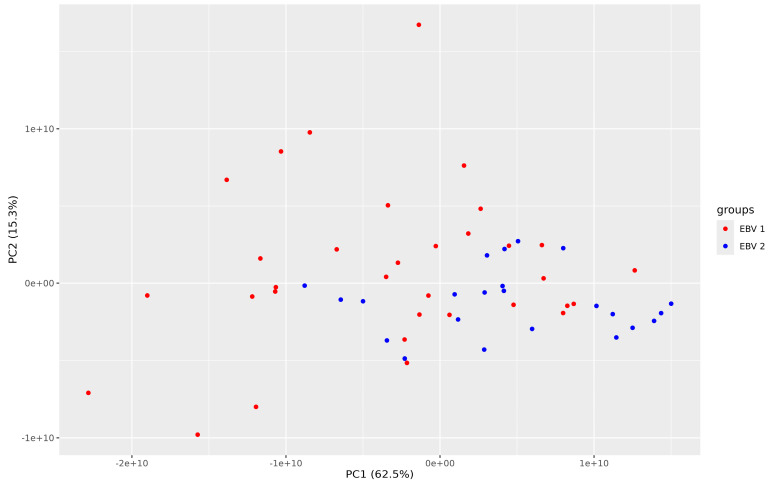
Principal component analysis (PCA) (N = 56 patients; K = 38 proteins), comparing groups EBV-1 vs. EBV-2.

**Figure 3:**
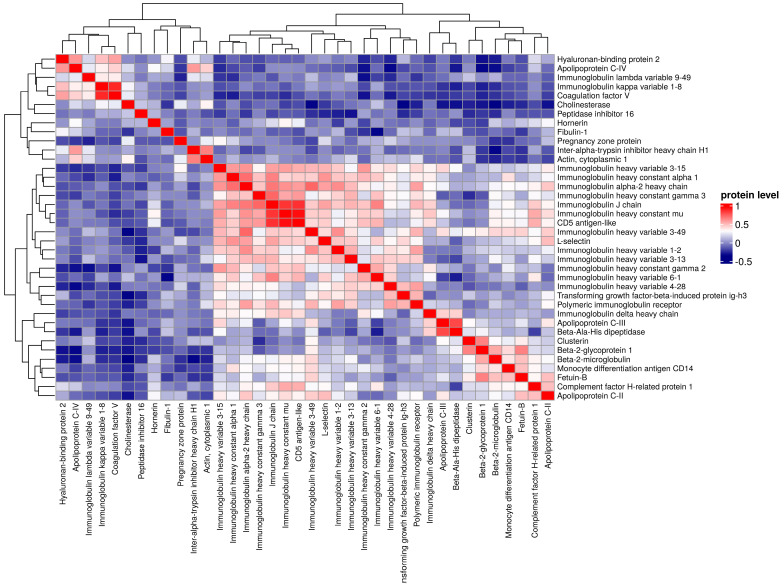
Pearson correlation analysis (N= 56 patients, K = 38 proteins), comparing groups EBV-1 vs. EBV-2.

A total of 38 proteins differentiating between EBV-1 and EBV-2 patients were identified. Among the acute phase proteins, EBV-2 patients had significantly higher levels of actin, lumican (LUM), peroxiredoxin-2, fibulin-1 (FBLN-1), gelsolin (GSN), hyaluronan-binding protein-2, and alpha-2-macroglobulin. Among other proteins, apolipoproteins A-I (APOA1), A-IV (APOA4), C-IV (APOC4), and M (APOM), as well as biotinidase (BTD), cholinesterase (BCHE), coagulation factor V, and the complement C1q, C1r, and C8 gamma (C8G) chain levels were significantly higher. The identified proteins were hierarchically clustered and presented as a heat map (Figure 1, Figure 2 and Figure 3; Supplementary Materials S1). 

The unattended PCA (principal component) analysis visibly ungrouped both experimental groups (Figure 2; blue circles—EBV-2 group, red circles—EBV-1 group). 

**Figure 4:**
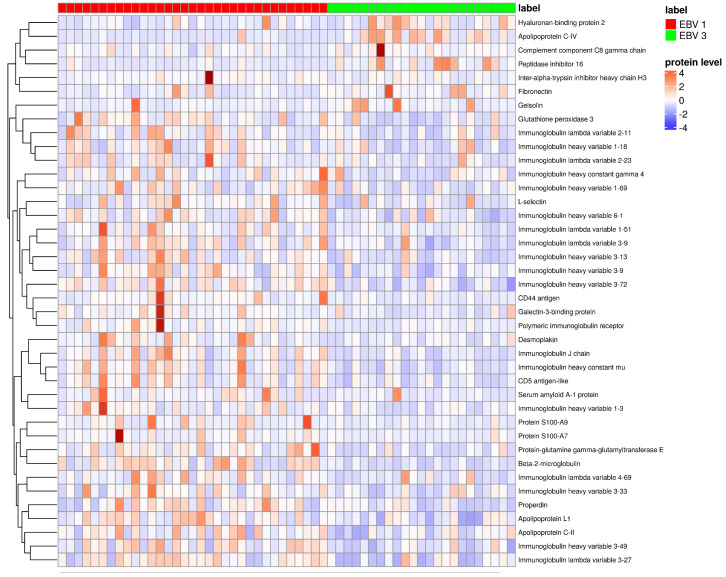
Hierarchical clustering of proteins (N = 56 patients; K = 40 proteins), comparing groups EBV-1 vs. EBV-3.

**Figure 5:**
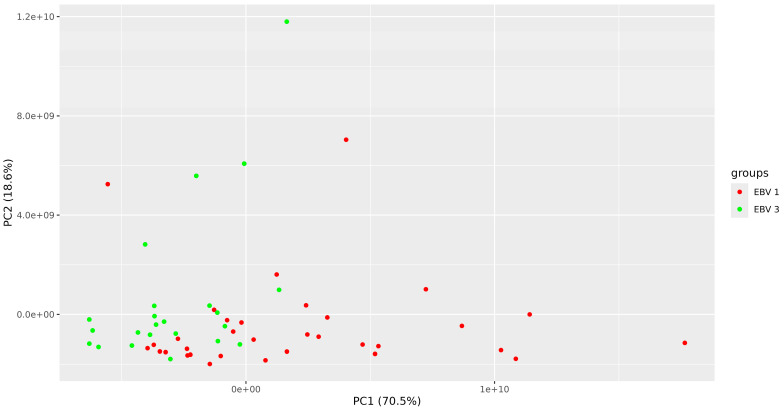
Principal component analysis (PCA) (N = 56 patients; K = 40 proteins), comparing groups EBV-1 vs. EBV-3.

**Figure 6:**
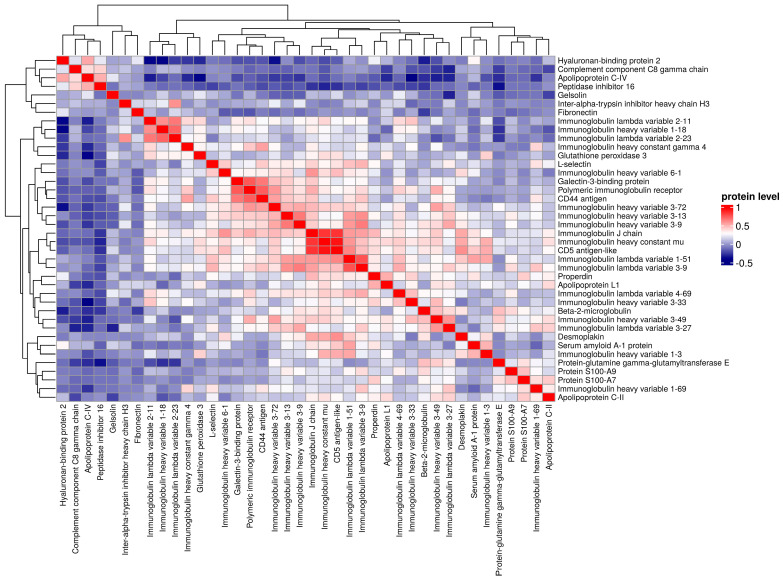
Pearson correlation analysis (N = 56 patients, K = 40 proteins), comparing groups EBV-1 vs. EBV-3.

A similar comparison was made between patients from the EBV-1 and EBV-3 groups. Forty differentiating proteins were identified (Figure 4, Figure 5 and Figure 6). Higher levels of serum albumin (ALB); apolipoproteins M (APOM), C-IV (APOC4), and A-I (APOA1); lumican (LUM); complement C8 gamma (C8G); fibronectin (FN1); attractin (ATRN); and complement C1r subcomponent-like protein (C1RL) were found in the plasma of convalescents. Levels of transforming growth factor-beta-induced protein (TGFBI), L-selectin (SELL), coagulation factor XII (F12), properdin (CFP), complement C2 (C2), galectin-3-binding protein (LGALS3BP), complement factor H (CFH), alpha-1-antichymotrypsin (SERPINA3), ceruloplasmin (CP), alpha-1-acid glycoprotein (ORM1), zinc-alpha-2-glycoprotein (AZGP1), complement C1s subcomponent (C1S), and glutathione peroxidase 3 (GPX3) were significantly lower (Supplementary Materials S1).

The unattended PCA analysis evidently ungrouped the EBV-1 vs. EBV-3 experimental groups (Figure 5).

**Figure 7:**
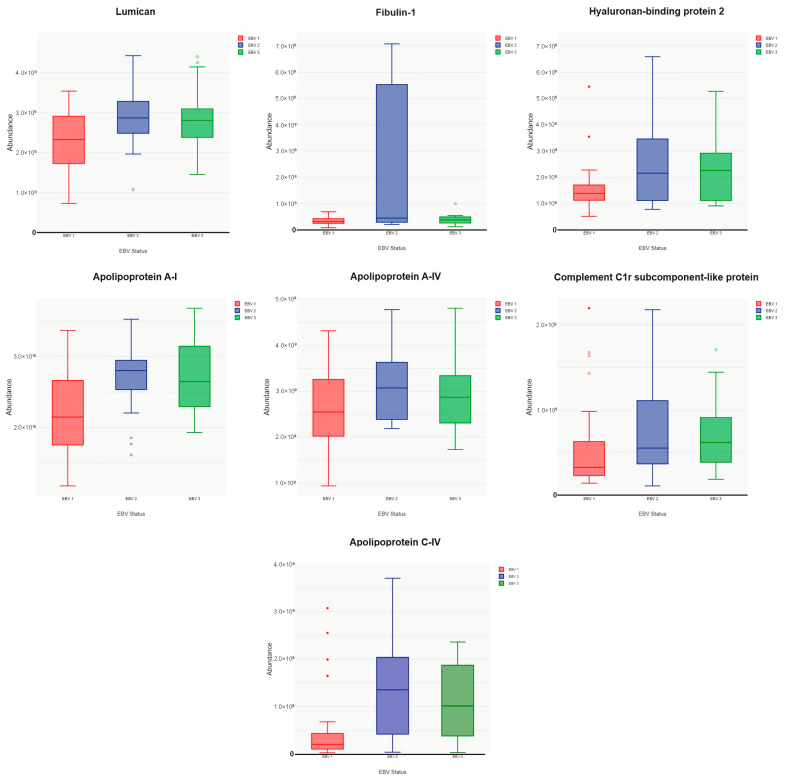
Comparative analysis of protein abundance, indicating its increase in the IM phase and persistence during the convalescent phase: statistical box plot with whiskers.

To gain more functional insight, we further analysed the differentially accumulated proteins in the context of associated biological processes (Figure 7). The enrichment test revealed that a response to stress was the most overrepresented process in both comparisons: EBV-1 vs. EBV-2 and EBV-1 vs. EBV-3. However, the number of dysregulated proteins involved in this process was different for both comparisons. We detected 24 stress proteins when EBV-1 and EBV-2 were compared, whereas 36 proteins functionally related to a response to stress were identified in the EBV-1 vs. EBV-3 comparison. 

In the next stage, we conducted a comparative analysis of the results obtained in three research groups as follows: naïve, patients with acute IM, and convalescents. As a result, we confirmed that during the acute EBV infection, there is an increase in the abundance of several proteins associated with the inflammatory process. The abundance of most of these proteins, which increased during the IM phase, decreases during the convalescent phase. Only three proteins, lumican, fibulin-1, and hyaluronan-binding protein 2, whose abundance increased during the acute phase, persist during the convalescent phase. A similar relationship was observed for three apolipoproteins: apolipoprotein A-I, apolipoprotein A-IV, and apolipoprotein C-IV. Among the complement components, only complement C1r exhibits persistent abundance during the convalescent period.

## 4. Discussion

EBV infection manifests itself as a wide range of symptoms in children. From asymptomatic forms through to the classic picture, including lymphadenopathy and hepatitis, to severe clinical forms involving various organs, including the brain [1] and the heart [3], the impact of EBV infection on the formation of tumours is also very well-known [5]. Infection passing into a latent form may become a cause of the neoplastic process in the future. The use of proteomic research allows the creation of proteomic maps [8]. 

Proteomics, as a methodological approach, encompasses the identification and characterisation of a repertoire of proteins present within a defined physiological or pathological context. Conducting proteomic comparative analyses among Epstein–Barr virus (EBV)-naive individuals and those exhibiting symptoms of acute infectious mononucleosis (IM) can elucidate distinct protein profiles associated with viral infection [8]. These proteomic profiles may encompass proteins present within viral particles, those interacting with specific viral proteins within host cells, or broader alterations in cellular protein expression within tissues affected by the infection. Continued monitoring of patients and conducting longitudinal comparative assessments in individuals experiencing the acute phase of infection, alongside those who remain asymptomatic, facilitates the evaluation of the virus’s enduring impact on the host. Our findings indicate significant alterations in the relative abundance of proteins within the proteome of IM patients. For analytical purposes, these proteins were categorised into distinct groups. Notably, within the acute phase protein group, heightened levels of actin, lumican, peroxiredoxin-2, fibulin-1, gelsolin, hyaluronan-binding protein-2, and alpha-2-macroglobulin were observed. Conversely, in individuals examined one year post-IM onset, sustained elevations were noted for lumican, fibulin-1, and hyaluronan-binding protein. These proteins play roles in B lymphocyte infection and apoptosis induction, albeit their precise functional significance remains incompletely understood. Nevertheless, their overarching involvement in the orchestration of inflammatory responses has been underscored [12,15,16,17,18].

The increase in actin levels may result from the viral killing of lymphocytes. As demonstrated by EBV studies, the virus selectively infects human lymphocytes with permissive membrane receptors. EBV enters the cell, which induces the proliferation and immortalisation of these cells [15]. One of the probable mechanisms is the stimulation of the conversion from globular actin to the filamentous form, a process that has been associated with the activation and transformation of the cells. Preincubation of B cells with botulinum C2 toxin or cytochalasin, which blocks the conversion of G-actin to F-actin, resulted in the inhibition of EBV-induced proliferation [18]. Treatment of EBV-positive cells with drugs that alter actin polymerisation specifically showed marked effects on splicing in this region. This suggests a potentially novel role for nuclear actin in the regulation of viral RNA splicing [19].

Fibulin-1 is a calcium-binding plasma and extracellular matrix (ECM) protein that has been implicated in cellular transformation and tumour invasion. It plays a role in cell adhesion and migration along protein fibres within the ECM and is important for certain developmental processes. Fibulin-1 contributes to the supramolecular organisation of ECM architecture, particularly that of the basement membrane [20]. It is implicated in cellular transformation and tumour invasion and can behave both as an oncosuppressor and oncogene depending on the tissue environment. This protein is involved in haemostasis and thrombosis, owing to its ability to bind fibrinogen and incorporate it into clots. It also plays a significant role in modulating the neurotrophic activities of amyloid beta precursor protein (APP), particularly the soluble form [21]. The importance of the increase in fibulin-1 in EBV infection has not been fully understood, although the protein’s influence in other viral infections, including oncogenic papillomavirus E6, has been confirmed [22]. 

The significance of the increase in lumican abundance in IM has not yet been clarified. It is an extracellular protein that associates with CD14 on the surface of macrophages and neutrophils and promotes a CD14-TLR4-mediated response to pathogens. According to the research by Maiti et al., lumican may play a dual protective role in barrier ECM tissues, one that promotes bacterial defence and another of limiting antiviral and autoimmune inflammatory responses [16]. Through a comprehensive review of the literature, it has been determined that the significance of lumican in relation to EBV infection remains unestablished.

Among the proteins isolated in the acute phase, the presence of markers of oxidative stress was also demonstrated. We confirmed an increased level of peroxiredoxin-2, which is one of the possible antioxidant mechanisms by which EBNA could induce reactive oxygen species (ROS) [23]. Enhanced production of ROS and dysbalanced antioxidant responses that are often referred to as oxidative stress were shown to be the hallmarks of many viruses from various families. They include HBV, HCV, HIV, various respiratory viruses, herpesviruses, etc. [24]. A recent study confirmed increased levels of ROS during in vitro and in vivo EBV infections in EBV-associated patients [25]. During a further observation of patients with EBV, no sustained elevation in the concentration of ROS factors was demonstrated.

Another category of proteins, isolated from the serum of paediatric patients diagnosed with IM, comprises apolipoproteins. Notably, apolipoproteins A-I, A-IV, C-IV, and M exhibited elevated levels in our analysis, with apolipoprotein C-IV displaying the most pronounced increase. Conversely, the abundance of apolipoproteins B-100 and C-II was found to be diminished in patients experiencing active IM compared to controls. This finding contrasts with prior studies; specifically, we observed a decrease in both apolipoproteins A-I and B-100 levels [25,26]. It is recognised that various viral and bacterial infections can induce alterations in the composition and functionality of lipoproteins. Existing evidence suggests that changes in the apolipoprotein concentrations during infection are integral to the acute inflammatory response and typically normalise approximately four months following symptom onset [27]. Diverging from the investigations referenced above, our analysis evaluated the protein accumulations post-acute IM phase, specifically 12 months following symptom onset. Intriguingly, our findings demonstrated that the apolipoproteins M, C-IV, and A-I levels remained elevated compared to the naive patients. This observation may signify a prior history of EBV infection in the child, akin to the CMV described in the literature, which serves as a risk factor for the subsequent development of coronary artery disease [28,29]. Complement system proteins, including C1q, C1r, and C8 gamma chain, play a crucial role in the immune response to acute IM, which is often caused by EBV infection. Therefore, an increase in their abundance in the acute phase of the disease remains justified. C1q is a component of the C1 complex, which is the first component of the classical pathway of the complement system. In the context of EBV infection and IM, C1q participates in the recognition of immune complexes formed by antibodies binding to viral antigens or infected cells. This recognition triggers the complement cascade, leading to opsonisation (marking of pathogens for phagocytosis) and activation of inflammation, which are essential for the clearance of viral particles and infected cells [21,30,31]. C1r is another component of the C1 complex, and it has a protease activity that is involved in the activation of the complement cascade. In our study, complement C1r is the only one showing an increase in abundance in the IM phase, and it maintains it after a year of convalescence. The significance of this increase has not yet been determined; however, in our study, upon binding of the C1q to immune complexes in EBV infection, C1r becomes activated and subsequently activates C1s. This activation initiates a proteolytic cascade, resulting in the generation of active complement components that contribute to opsonisation, recruitment of immune cells, and lysis of infected cells [30]. The C8 protein is a component of the terminal complement pathway. It forms a complex with other complement proteins, including C8 alpha and C8 beta chains, to create the membrane attack complex (MAC). The MAC is responsible for inserting into the membrane of target cells, such as infected cells, leading to cell lysis. In the context of EBV infection, the terminal complement pathway can contribute to the destruction of EBV-infected cells [32,33].

The study carried out is of a pilot nature. The authors are aware of the limitations of this study related to the small number of subjects and the imperfection of serological diagnosis of EBV infection. It should be emphasised, however, that the analysis was performed in a hospital centre, where in clinical practice, it is necessary to confirm symptomatic EBV infection using serological tests. PCR-based diagnostics are reserved for patients with primary and secondary immunodeficiencies and in doubtful cases. It includes a group of patients rarely analysed in proteomic studies, namely the paediatric group. Children and adolescents are particularly vulnerable to illnesses caused by EBV infection, most of which are symptomatic. This creates a special opportunity to examine patients at the peak of their clinical symptoms. The presented protein shifts at the peak of EBV infection and after IM may become the basis for further research on the impact of the virus on the further development of children.

## Figures and Tables

**Table 1:** The characteristics of the examined group with division into research subgroups.

	EBV-1	EBV-2	EBV-3
n	33	23	23
Mean age (years)	8.57 ± 3.56	9.09 ± 2.53	10.13 ± 2.44
Female/male	12/21	11/12	11/12
*p*	0.533		

## Data Availability

The datasets used and/or studied during the current study are available from the corresponding author upon reasonable request.

## Supplementary Materials

The following supporting information can be downloaded at: https://www.mdpi.com/article/10.3390/pathogens13060471/s1, Supplementary Materials S1. Mean abundance of proteins in research groups, *p*-value, and fold change.
