# Supplementary material for: The Impact of Acute EBV Infection on Changes in the Serum Proteome in Children—A Pilot Study
Source: Pathogens. 2024 Jun 4;13(6):471. doi: 10.3390/pathogens13060471 (PMC11206626; doi:10.3390/pathogens13060471)
Supplement: Supplementary file 1 [file pathogens-13-00471-s001.zip › pathogens-2972577-supplementary.pdf]

Supplementary materials 1. Mean abundance of proteins in research groups, p-value, and fold change

Protein name	EBV -1 (Mean abundance)	EBV-2 (Mean abundance)	EBV-3 (Mean abundance)	EBV-1 /EBV-2 p-value	EBV-1 /EBV-2 Fold change	EBV-1 /EBV-3 p-value	EBV-1 /EBV-3 Fold change
Beta-2-glycoprotein 1	428200921,2	1126981213	143236548,9	0,000016042	2,781082933	0,000355896	1,544952659
Beta-2-microglobulin	423579281	26720756,4	3873014,064	0,000170447	2,028129267	0,001823827	2,761713939
Monocyte differentiation antigen CD14	154654663,9	5873118,4	14636869,15	0,007898268	1,937294081	0,001918374	1,597253456
L-selectin	229369481,3	24680576,5	212181208,5	0,000567595	1,895369356	0,003615442	1,453185561
Pregnancy zone protein 04	3003896454	37835397,1	5988158,251	0,000660822	1,891407315	0,009391864	2,029305388
Polymeric immunoglobulin receptor	166412700,6	41743352,5	14716222	0,002045967	1,835798408	0,015289686	1,684686399
Transforming growth factor-beta-induced protein ig-h3	9127593813	13269152,4	24436971,72	0,04694922	1,825930866	0,015837394	1,605960715
Immunoglobulin heavy variable 4-28	1552205520	27411826,1	12112047,46	0,000595862	1,809764672	0,026117203	1,811295395
Fetuin-B	374102628,8	23616189,6	162427121	0,002454794	1,730128103	0,027470552	1,565329644
Immunoglobulin heavy variable 3-15	409680451,4	310538042,4	138378425,5	1,7855712867	1,697835348	0,043931174	1,294758117
Immunoglobulin delta heavy chain	139785480,8	127865951,5	14735326408	0,004414446	1,684245413	0,013877749	1,205309417
Beta-Ala-His dipeptidase	24792219,1	12637694,6	20892599,55	0,009950278	1,680138944	0,038183815	1,446979359
Immunoglobulin J chain	8217871290	439707093,6	96081635,68	0,005163273	1,626007717	0,000044274	0,155325486
CD5 antigen-like1	324798059,2	455074649	24477530	0,000858289	1,611314551	0,000047363	2,213995781
Immunoglobulin heavy constant mu	807057953,9	6721371581	103350491,3	0,000898085	1,594544942	0,0009591	1,505289549
Clusterin OS	17760627681	2480722705	297193305	0,010443169	1,589751171	0,001024655	1,440816176
Immunoglobulin heavy constant alpha 1	69758011454	9986658682	99860496,52	0,013522719	1,569385492	0,001397205	1,484650646
Immunoglobulin heavy variable 3-13	146246770,1	15358411,3	198615404,9	0,003806698	1,522214076	0,01109034	1,362132754
Immunoglobulin heavy variable 3-10	155572414,4	97821681,2	111292474,1	0,000664186	1,515593975	0,031004825	1,622400732
Immunoglobulin heavy constant gamma 2	70704536,9	8001127135	89469732,96	0,000214513	1,509533163	0,000025076	1,562377312
Immunoglobulin alpha-2 heavy chain	178521910,9	555782174,6	19073516,66	0,00167207	1,476004357	0,001776276	1,796492256
Immunoglobulin heavy variable 6-	507461668,4	292266635,5	31751557,93	0,001097226	1,465103673	0,012532692	1,473276003
Apolipoprotein C-III	2893423465	290093514,4	423528993,4	0,029928368	1,460147366	0,026608023	1,244879273
Complement factor H-related protein 1	221293687	105991081,4	13363849,81	0,001968977	1,459129032	0,030500308	1,681451937
Apolipoprotein C-II	206118113,2	158629200,8	146991457,6	0,005477006	1,445947406	0,045820221	2,176549566
Immunoglobulin heavy constant gamma 3	25848134,3	2115763111	199200874,1	0,022757637	1,419769746	0,000935232	1,28849871
Immunoglobulin heavy variable 1-2	21976418304	118675878,5	6951079480	0,041845341	1,402245365	0,00195268	1,313118896
Immunoglobulin lambda-1 light chain	2540203216	6570573813	16640209,34	0,000377031	1,389162358	0,004509794	3,022307811
Immunoglobulin lambda constant 3	42736828540	1121526186	159836029	0,001999853	1,38401184	0,009757489	1,435029903
Complement factor I	227641548,4	271824236,5	256065341,2	0,004798256	1,376266641	0,032189534	1,267452368
Immunoglobulin kappa variable 3-11	13803435,2	299911879,7	362542827,8	0,003866655	1,366002747	0,000097164	1,972090116
Immunoglobulin heavy variable 3-72	214044941,6	102661145	21964057,98	0,001657094	1,361620123	0,033209952	1,721124711

Supplementary materials 1. Mean abundance of proteins in research groups, p-value, and fold change

Immunoglobulin lambda variable 2-11	13686052,3	18292866,5	36397567,65	0,016711563	1,355294373	0,000051787	0,174836694
Apolipoprotein B-100	39252354,12	6074439880	5968771,389	0,003698553	1,35286075	0,003434585	3,474111728
Immunoglobulin kappa variable 4-1	155485158,4	248764753,7	600405327,3	0,00045872	1,305643401	0,016111348	1,366305184
Immunoglobulin kappa variable 3D-7	1504339472	621015431	69960858,61	0,002961677	1,299577939	0,042096584	0,770399186
Immunoglobulin kappa light chain	5854868,981	13691044909	29296148,28	0,001596865	1,297244133	0,04673813	1,394689597
Immunoglobulin gamma-1 heavy chain	457410012,2	53958110074	5873083336	0,000153866	1,292817917	0,000032698	1,824855607
Immunoglobulin kappa variable 1-17	111733942,3	113852953,9	265890448	0,039921279	1,284523282	0,004311436	0,805011775
Immunoglobulin heavy variable 3-9	6363630,4	121689264,2	4822430208	0,039559798	1,27843993	0,004924887	1,360706506
Apolipoprotein L1	14923926,78	55311558,3	1198748953	0,025768205	1,278295877	0,036511650	1,413171888
Insulin-like growth factor-binding protein complex acid labile subunit	19166531,4	139698384,7	3018935,531	0,042720546	1,277909629	0,000118150	2,576168217
Protein AMBP	33545212,1	398569524,2	120716934,8	0,015677344	1,273207402	0,023734338	0,693773944
Immunoglobulin kappa constant	428200921,2	2303394399	288224806,3	0,008172235	1,256156335	0,004462786	0,789805539
Immunoglobulin lambda variable 3-27	423579281	181944041,4	234685331,1	0,034887888	1,216273341	0,011298416	0,662526105
Thyroxine-binding globulin	154654663,9	178883152,3	46004245,42	0,023181345	1,152250005	0,001185453	1,536913306
Biotinidase	229369481,3	31518555,4	82057087,13	0,043806368	0,820092608	0,003083225	1,478827827
Apolipoprotein A-I	3003896454	26878894926	170784488,7	0,001551821	0,817608699	0,014635892	1,206890127
Apolipoprotein A-IV	166412700,6	3110065096	27000204,6	0,011943493	0,816768504	0,001706592	2,838214793
Alpha-2-macroglobulin	9127593813	53198896440	58497901791	0,000251963	0,803340509	0,005252740	1,192487411
Lumican	1552205520	291454418,8	672299936	0,005087527	0,781053687	0,024067875	1,141533761
Peroxiredoxin-2	374102628,8	18922208,5	443720140,5	0,012542975	0,729483305	0,000436148	1,652547038
Apolipoprotein M	409680451,4	297301063,7	293566608	0,000587389	0,719960228	0,006183015	1,317969809
Immunoglobulin lambda variable 9-49	139785480,8	20101949,87	170043082,1	0,028846339	0,68083208	0,024078234	1,358848946
Cholinesterase	24792219,1	58888642,4	637244514,8	0,009710973	0,666552199	0,023658986	1,558142944
Hyaluronan-binding protein 2	8217871290	257720914,8	3084723,895	0,008107223	0,603308267	0,020620247	1,918269048
Inter-alpha-trypsin inhibitor heavy chain H1	324798059,2	2704694211	203191127,7	0,002013786	0,556195767	0,023416708	1,241302395
Hornerin	807057953,9	10890085,3	174180177,4	0,021412943	0,537632976	0,040743918	0,856294715
Actin, cytoplasmic 1	17760627681	1009348858	2611827534	0,016517738	0,453173359	0,013373215	1,293516901
Immunoglobulin kappa variable 1-8	69758011454	275435433	1325283981	0,016802662	0,405662921	0,039075118	0,609086979
Peptidase inhibitor 16	146246770,1	16434118,4	27282431288	0,042312228	0,387220673	0,000630499	0,805515391
Apolipoprotein C-IV	155572414,4	95242957,33	3664264604	0,000502307	0,156693232	0,016611892	1,172978589
Coagulation factor V	70704536,91	389220637	3855996294	2,16E-05	0,049243358	0,011849673	1,192833068
Fibulin-1	178521910,9	766228125	3102081842	0,000457954	0,043779667	0,011921402	0,590348048

Legend: EBV 1- EBV-1, (n=33): the group included material obtained from healthy patients; EBV 2 - EBV-2, (n=23): material from patients presenting symptoms of acute infectious mononucleosis; EBV-3 (n=23): after IM, the children from group EBV-2 were observed over the next 12 months.
